# Linking Sexting Expectancies with Motivations to Sext

**DOI:** 10.3390/ejihpe12020016

**Published:** 2022-02-09

**Authors:** Joseph M. Currin

**Affiliations:** Department of Behavioral Sciences and Leadership, United States Air Force Academy, USAF Academy, CO 80840, USA; joseph.currin@afacademy.af.edu

**Keywords:** sexting, sexting motivations, sexting expectancies

## Abstract

While many researchers have explored the impact sexting may have on relationships and mental health, few have explored the motivations and expectancies as to why individuals engage in sexting. By understanding why individuals sext their partners, we can learn more about what drives the behavior. Therefore, the current study sought to determine if sexting for sexual purposes (SP) or body image reinforcement (BIR) would predict positive sext expectancies. There was no prediction for instrumental/aggravated reasons (IAR). The online questionnaire had 348 participants, and based on regression analysis, positive sext expectancies while sending a sext message predicted sexting for sexual purposes. Somewhat surprisingly, sexting for instrumental/aggravated reasons was predicted by negative sext expectancies (both sending and receiving). These findings demonstrate individuals who sext for sexual purposes, and have positive sext expectancies, appear to enjoy the consequences of that behavior. Individuals who sext for instrumental/aggravated reasons may be uncomfortable with the outcome of their sexting behavior. This result highlights an area where clinicians could help clients explore the true reinforcements behind IAR.

## 1. Introduction

While various definitions of sexting have been established, most often sexting is defined as the sending and/or receiving of a sexually suggestive or explicit text, partial nude photo or video with sexual conations, or a fully nude photo or video [1]. Sexting a partner has become a somewhat normative behavior in modern American society, with studies demonstrating that over half of a sample engaged in this behavior [2] as well as in other countries including Australia [3], Spain [4], and Germany [5]. Sexting behaviors have been associated with potential mental health outcomes [6,7,8], associated with other sexual behaviors like multiple partners and condomless sex [9], and certain emotional experiences when sexting [1]. One emerging area of research in regards to sexting behavior is the study of why individuals participate in sexting. By exploring both the motivations and consequences to sext, we can better understand why people engage in this behavior.

With research exploring both the negative and positive aspects to sexting [2,10], understanding the motivations and the expectancies associated with sexting are important nuances for clinicians and others working with individuals who participate in sexting. Individuals who engage in sexting behaviors may view the behavior as fun and exciting [1] or even as a confidential way to express their sexuality [11,12], and thus report the behavior as beneficial or enhancing their romantic/sex lives. However, others may experience negative outcomes to their sexting behaviors, but still engage in sexting. Understanding what continues to motivate a person to sext even when expecting a negative outcome would help highlight why an individual continues to participate in a behavior that causes potential distress.

### 1.1. Sexting Motivations

Humans engage in sexual behaviors for more than biological drives, as first postulated by Freud (1955) [13]. Hardy (1964) [14] built upon Freud’s drive theory by highlighting how humans engage in sexual behavior for reasons other than procreation, specifically that each person contains a desire or appetite for sex, which he called appetitional theory. Appetitional theory expands on the biological drive to engage in sexual behavior by exploring the cognitive and affective motivations that exist as well [15]. Further research by Meston and Buss identified 237 reasons individuals engage in sexual behavior, ranging in grouped motivations of pleasure and reproduction to mate guarding and duty/pressure [16,17]. With wide ranging motivations to engage in sexual behaviors, one would expect similar types of reasons to engage in sexing behavior.

As sexting is a sexual behavior, it logically follows then that there are affective and cognitive motivations that exist for engaging in sexting behavior. Motivations of sexting have been studied in both adolescents [18,19] and adults [12]. As originally described by Bianchi and colleagues [18], individuals sext for three main reasons: sexual purposes, body image reinforcement, and instrumental/aggravated reasons. Individuals who engage in sexting for sexual purposes are doing so for the ultimate goal of having sex with the person(s) whom they are sexting [18]. Individuals who engage in sexting for body image reinforcement are often dealing with anxiety about their appearance or status in the current relationship, and therefore sext to receive reassurance [1,19,20,21]. Finally, individuals also are motivated to sext for more instrumental or aggravated reasons, which describes a collection of motivations that indicate a person is sexting for a reason other than wanting to have sex with the person whom they are sexting and/or they are not wanting to receive reinforcement about their body image. Instrumental/aggravated reasons to engage in sexting include activities like sharing sexts without the permission of the sender (an example of aggravated motivations) or participating in sexting even though the person does not necessarily want to sext (an example of an instrumental reason [18,22,23]. Motivations related to IAR to sext are similar to the study by Meston and Buss (2007) [16] that highlighted various reasons individuals engage in sex, including utilitarian motivations which included aspects such as to gain favor with someone, or reward a person for paying for an expensive dinner. While these motivations deal with some of the cognitions and/or emotions associated with the antecedents of sexting behavior, the expectancies (or consequences) of engaging in sexting are equally important in influencing this behavior.

### 1.2. Sexting Expectancies

Behavior is influenced by not only the antecedents to that behavior, but also by the consequences [24]. Expectancy theory, first articulated by Vroom [25] and associated with decisions individuals make at work, expands on Skinner’s view that consequences influence our behaviors. Expectancies are associated with both the valence of the outcome as well as the belief that if a person engages in a behavior, the believed outcome will occur. The valence of the outcome is the emotional evaluation of the outcome, often labelled as positive or negative [26]. The belief that an outcome will happen if a person engages in that behavior is known as an expectancy. Expectancies have been explored with alcohol use [27], condom use [28], and even substance use [29]. 

Individuals who sext also have expectancies on the outcome of the behavior they are participating, called sexting expectancies [30]. Sexting expectancies have been explored by behavior (sending and receiving) [3,31] as well as by valence (positive and negative) [1,30]. Individuals who have positive sext expectancies believe that sexting makes one more affectionate, intimate, more likely to have sex, and even more attractive to others [30]. This is similar to findings that individuals who sext feel more excited, connected, and loved [1]. Negative sext expectancies, however, are the belief that engaging in the behavior will make the person feel disgusting, shameful, and inappropriate [30]. Interestingly, while we know motivations to sext and expectancies people have about sexting, few researchers have studied how these variables are linked.

### 1.3. Current Study

Understanding sexting expectancies and how they relate to sexting motivations contributes to a better understanding of why individuals engage in this behavior. Specifically, to add to the literature about why individuals sext, I aimed to explore how sexting motivations (an antecedent to the behavior) are related to sexting expectancies (the evaluation of the expected outcome of the behavior). In line with expectancy theory, I hypothesized that individuals who engage in sexting for sexual purposes or body image reinforcement will have a positive sext expectancy, meaning they believe a positive outcome will take place after sexting (e.g., feeling attractive, desired, and more likely to have sex). There was no prediction for individuals engaging for instrumental/aggressive reasons due to a lack of information on how individuals evaluate the outcome of sexting for these reasons.

## 2. Materials and Methods

Participants were recruited on Amazon’s Mechanical Turk (MTurk) website if they met the following criteria: lived in the continental United States, were 18 years of age or older, had sent and/or received a sext message in the last 12 months, and had successfully completed MTurk tasks before accepting to participate in this study. Individuals responded to the request on MTurk that stated “Participants wanted to explore influences related to motivations to sext.” Participants who accessed the link were presented with the informed consent documents, and if they provided consent they proceeded to the online questionnaire and completed measures on motivations to sext, sexting expectancies, and demographic information. The online questionnaire was originally accessed by a total of 427 individuals, but 42 individuals were removed due to providing incomplete data or failing the manipulation checks. This left a total of 385 individuals included in the current study. Participants received USD 2 credit to their MTurk accounts for completing the study, and the study took around 10 min to complete. All methods were approved by the Institutional Review Board at Texas Tech University.

### 2.1. Materials

#### 2.1.1. Participant Validation

To ensure that individuals who accessed the study link were focused on the items presented, instructional manipulation checks (IMCs) were used throughout the survey. Using passive and active IMCs helps to ensure participants are thoughtfully responding to the items on the questionnaires [32]. An example of a passive IMC is having an item embedded with a questionnaire that states “Please select ‘often’.” An example of an active IMC is asking the participant to type three colors into a blank field. Participants who were unable to successfully execute the IMCs were removed from the study.

#### 2.1.2. Sociodemographics

Participants provided their race/ethnicity from the following: Asian/Pacific Islander, American Indian/Alaskan Native, Black/African American, Latino/Latina, White–not of Hispanic origin, Another Race/Ethnicity not listed, Biracial/Multiracial, Decline to Answer. Gender was identified by participants from the following: male, female, transgender (male to female), transgender (female to male), not listed (please specify). Participants identified their sexual orientation from the following list: heterosexual, mostly heterosexual, bisexual, mostly gay/lesbian, gay/lesbian, asexual, not listed. Relationship status was identified by participants from the following list: in a committed relationship (not living together), in a domestic relationship (living together), married, in an open relationship, in a consensual non-monogamous relationship, dating, other (please define), and single. Participants also provided their age in years.

#### 2.1.3. Motivations to Sext

The sexting motivations questionnaire (SMQ) was designed to assesses three different types of motivations that individuals may have when sexting: sexual purposes (SP), body image reinforcement (BIR), and instrumental or aggravated reasons (IAR) [18]. The measure has been used in adolescent, emerging adult, and established adult populations with Cronbach’s α of 0.76 or higher for all subscales [12,18]. In the current study, Cronbach’s α were 0.82 or higher for each subscale. Examples of items on the SMQ are “Sometimes I send sexts to increase passion in my dating relationship,” and “Sometimes I send texts to test whether I am attractive enough” and are rated on a Likert-type scale from 1 (Never) to 4 (Always) [18]. Scores were calculated for each subscale by adding the responses for the subscale and then dividing the subscale by the number of items included in the subscale. Higher scores on a particular subscale indicate a stronger motivation to engage in sexting (e.g., higher scores on SP subscale indicates stronger motivations to sext for sexual purposes).

#### 2.1.4. Sexting Expectancies

The Sextpectancies Questionnaire (SQ) was used to determine the positive and negative expectancies that individuals may have when engaging in sexting behaviors [30]. The SQ has four subscales, measuring positive sext expectancies when sending (PSES) and receiving (PSER) sext messages as well as negative sext expectancies when sending (NSES) and receiving (NSER) sext messages. The SQ measures items on a Likert-type scale from 1 (not true at all) to 4 (extremely true). Examples of some items on the SQ are “Receiving sexts makes one feel sexy,” and “Sexting makes one feel vulnerable.” The Cronbach’s α for the original sample were 0.85 or higher [30], and similar Cronbach’s α were observed in the current study (0.84 for PSER, 0.91 and higher for all other subscales). Scores were calculated for each subscale by adding the responses for the subscale and then dividing the subscale by the number of items included in the subscale. Higher scores on PSES and PSER indicate a stronger expectation that a positive outcome would follow if the person sends and/or receives a sext message; similarly higher scores on the NSES and NSER indicate a stronger expectation that a negative outcome would follow.

### 2.2. Data Analysis Plan

To predict which motivations to sext (SP, BIR, IAR) are based on which expectancies (PSES, PSER, NSES, NSER), a bivariate correlation was conducted between each motivation to sext and each sext expectancy subscale to determine which subscales should be included in the regression analysis. Each significantly correlated sext expectancy subscale was included in the regression to determine what significantly predicts the SP, BIR, and IAR subscales of the sexting motivations questionnaire. There were separate linear regressions conducted for each subscale of the sexting expectancy scale to determine which expectancies best predict the motivation to participate in sexting. Furthermore, the regressions were separated by gender to determine if there are any differences in motivations based on gender, as previous research has shown different experiences with sexting based on gender [12,18]. Therefore, a total of eight regressions (one for each of the four subscales for men, and one for each of the four subscales of women) were performed. To be conservative, a Bonferroni correction was applied to the significance level, so in order for an outcome to be considered significant the *p* value had to be less than 0.006.

For all eight of the regression equations, collinearity was assessed using the variance inflation factor (VIF). A value of five or higher for a predictor indicates high multicollinearity. Conservatively, if VIF is less than 2.5 for a predictor then collinearity is not a concern for regression results [33,34]. In the current study, the VIF values for men were 2.39 for BIR, 2.29 for IAR, and 1.07 for SP, and for women the VIF values were 1.58 for BIR, 1.55 for IAR, and 1.03 for SP. Therefore, collinearity is not a concern with the current study.

## 3. Results

### 3.1. Participant Sociodemographics

Out of the 385 participants in the current study, the majority identified as White, not of Hispanic origin (268, 69.6%), male (216, 56.1%), heterosexual (298, 77.4%), and that they were currently in some type of relationship including but not limited to marriage, domestic partnership, open relationship, triad, a consensual non-monogamous relationship, and dating (358, 93.0%). Participants reported a mean age of 30.4 years (SD = 9.5) with a range of 18 to 69 years of age.

### 3.2. Multivariate Analysis

Bivariate correlations (See Table 1) demonstrated that the subscales of the sexting motivations questionnaire are significantly correlated with the sexting expectancies questionnaire. Specifically, SP was significantly correlated with PSES (r = 0.56, *p* < 0.001) and PSER (r = 0.40, *p* < 0.001); IAR was significantly correlated with NSES (r = 0.66, *p* < 0.001), PSER (r = 0.13, *p* = 0.009), and NSER (r = 0.65, *p* < 001); and BIR was significantly correlated with PSES (r = 0.12, *p* = 0.015), NSES (r = 0.51, *p* < 0.001), PSER (r = 0.20, *p* < 0.001), and NSER (r = 0.50, *p* < 0.001).

#### 3.2.1. Positive Sexting Expectancies

For both men and women, the regression equations for PSES and PSER were significantly predicted by sexting motivations. For men’s positive sext expectancies when sending FPSES (3, 212) = 27.92, *p* < 0.001, R2 = 0.283 with one significant motivation predictor, SP (β = 0.54, t = 8.97, *p* < 0.001), and for receiving sext expectancies FPSER (3, 212) = 11.24, *p* < 0.001, R2 = 0.137 with one significant motivation predictor, SP (β = 0.32, t = 4.82, *p* < 0.001). For women’s positive sext expectancies when sending FPSES (3, 162) = 31.51, *p* < 0.001, R2 = 0.369 with one significant motivation predictor, SP (β = 0.58, t = 9.20, *p* < 0.001), and for receiving sext expectancies FPSER (3, 162) = 16.23, *p* < 0.001, R2 = 0.231 with one significant motivation predictor, SP (β = 0.45, t = 6.36, *p* < 0.001). Results indicate that if a person is sexting their partner for the purpose of having sex with them, they have positive sexting expectancies when sending and receiving sext messages. They judge the outcome of their behavior (sexting) as positive.

#### 3.2.2. Negative Sexting Expectancies

For both men and women, the regression equations for NSES and NSER were significant demonstrating that negative sexting expectancies can be predicted by sexting motivations. For men’s negative sext expectancies when sending FNSES (3, 212) = 67.27, *p* < 0.001, R2 = 0.488 with one significant motivation predictor, IAR (β = 0.60, t = 8.02, *p* < 0.001), and for receiving sext expectancies FNSER (3, 212) = 72.26, *p* < 0.001, R2 = 0.506 with one significant motivation predictor, IAR (β = 0.58, t = 7.92, *p* < 0.001). For women’s negative sext expectancies when sending FNSES (3, 162) = 22.82, *p* < 0.001, R2 = 0.297 with one significant motivation predictor, IAR (β = 0.50, t = 6.12, *p* < 0.001), and for receiving sext expectancies FNSER (3, 162) = 24.68, *p* < 0.001, R2 = 0.314 with two significant motivation predictors, SP (β = −0.23, t = −3.46, *p* = 0.001) and IAR (β = 0.48, t = 5.87, *p* < 0.001). Results indicate that if a person is sexting their partner for instrumental/aggressive reasons, they have negative sexting expectancies when sending and receiving sext messages. Furthermore, for women, sexting for the motivation of sexual purposes is negatively associated with predicting negative sexting expectancies when receiving a sext message.

Taken together, these results highlight the complexity behind sexting behaviors. Regardless of gender, when individuals participate in sexting for the purpose of having sex with their partner, they judge the outcome of the sexting behavior as positive. When people sext a partner for instrumental/aggressive reasons, they judge the outcome of the sexting behavior as negative. 

## 4. Discussion

The purpose of the current study was to determine if sext expectancies (positive or negative) could be predicted by sexting motivations (sexual purposes, body image reinforcement, and/or instrumental/aggressive reasons). The hypothesis that positive sexting expectancies were related to the sexting motivations of sexual purposes and body image reinforcement was partially supported. Specifically, positive sexting expectancies (both sending and receiving) were predicted by having the motivation to sext due to sexual purposes. However, body image reinforcement did not predict any type of sexting expectancy. Furthermore, an unexpected relationship was found between negative sexting expectancies and instrumental and aggravated reasons to sext. Specifically, IAR motivations to sext were associated with negative sexting expectancies, both sending and receiving.

The finding that positive sexting expectancies while sending and receiving a sext message for both men and women predicted sexting for sexual purposes is in line with previous research on sexting. The most common motivation cited in literature for sexting behaviors is sexting for sexual purposes [12,18]. Previous research has also provided qualitative themes of feeling excited, loved, connected, and mischievous when sexting a relationship partner [1]. With the antecedent of sexting being motivated to sext for sexual purposes, it would logically make sense that the sender would then expect the consequence to sexting to be engaging in a sexual relationship with the receiver. 

While there was no expectation that sexting expectancies would predict sexting for instrumental and aggravated motivations, negative sending and receiving sexting expectancies predicted IAR motivations to sext. This finding is truly novel in the sense that previous research has not explored the emotional responses or expectations of those who sext due to IARs. Bianchi and colleagues [18] defined sexting motivations related to IAR as “…sexting for secondary aims, not related to sexuality (e.g., for obtaining money, gifts, or small favours related to the perpetration of, and victimisation by violence), (p. 9).” Due to sexting being an actual antecedent to another behavior, in this case to gain favors and/or to victimize an individual, logically sexting expectancies would be unrelated to this motivation. Negative sexting expectancies related to IAR motivations to sext might be highlighting how an individual may feel some negative emotions in regards to using sexting for secondary reasons. For example, in a previous study [1] participants shared feelings of guilt, shame, and/or dirty feelings when sexting their partners. The negative expectation of an outcome for IAR motivations may also be alluding to the impact on the relationship itself. Individuals who sext for IAR may be gaining something in the short term, but they expect over the long term the relationship may deteriorate. More research into this unique finding is warranted to determine how negative sext expectancies are related to the motivation of IAR to sext.

Individuals who engage in sexting have been shown to rate higher on objectification measures [35]. Those who engage in self-objectification often experience negative emotions surrounding the behavior, including shame, vulnerability, and uncomfortableness in sharing the images [36,37,38]. Interestingly though, for both men and women, neither negative or positive sexting expectancies were predicted by body image reinforcement reasons for sexting, regardless of whether a person was sending or receiving a sext message. This finding is somewhat unexpected, as previous research has documented that individuals will send nude or sexually suggestive images to partners to receive feedback about their appearance [12,19]. This is surprising in that individuals who sext for BIR may be doing so due to desiring affirmation of their appearance. One would then assume they would receive reinforcement for this behavior from the target, or provide reinforcement if they received such a message, and thus develop an expectation on the outcome in participating in sexting. This may have not been detected due to the high number of individuals who were in a relationship, and may not use sexting as a means to gain reinforcement about their body image.

### Limitations

As in all studies, some limitations to the finding do exist. First, the sample was not random, but rather an online convenience sample of individuals. This may impact the external validity because those who participated in the online study may be more apt to engage in other online behaviors, like sexting, and therefore not representative of the population as a whole. As a convenience sample collected online, the results may not reflect the experiences of all subgroups of the sample. MTurk users tend to be slightly younger than the U.S. population (30 vs. 36 years of age), more identify as female (69% vs. 51% of national population) and more educated (63% college educated vs. 25% of the population), MTurk is more likely a representation of the population using the internet in the U.S. [39]. However, MTurk has been useful in the recruitment of populations when the subject matter may be stigmatizing (like sexual behaviors) and anonymity is desired by participants [40]. 

Another issue involving the sample and study design is that the study design was cross-sectional. Causation cannot be drawn from a convenient sample and from a cross-sectional design. Future research would benefit from researching aspects on sexting from a longitudinal approach and to use a random sampling technique. The consent to engage in sexting was not assessed in the current study. Some individuals may have received sext messages and did not consent to receive sexts. In previous work, the receipt of unwanted, consensual sext messages was correlated with negative outcomes, and this may be a reason for some individuals to evaluate receiving sext messages with negative sext expectancies. In their experience, the receipt of a sext message may be associated with feeling victimized [41]. Future researchers need to inquire about consent to sext to ensure all individuals had consented to the behavior. The current study did not differentiate who the individuals may be participating with when sexting, mainly whether it was their relationship partner or someone else (e.g., casual acquaintance, person they just met etc.). The relationship with these individuals may influence the motivation. In the current study, 93% of individuals indicated being in a relationship with someone else, and the assumption made was that they were sexting their relationship partner(s). Future research would benefit from understanding how motivations to sext may change based on relationship status.

Researchers have yet to agree on a universal definition of what constitutes sexting behavior [42], however the definition used in the current study has been used in a variety of studies by multiple researchers [18,19,35,43,44,45]. The main concern is that while various researchers may operationalize the sexting differently, however sexting is defined needs to be shared with the participants, as was the case in the current study. 

## 5. Conclusions

These limitations notwithstanding, the findings in the current study help expand what we know about the motivations and expectancies people have when sexting. Sexting behaviors within the literature have been labelled as both problematic and as a normal part of human sexual expression. The current study highlights how both of these seemingly opposite labels may be true. These results add to the understanding of sexting by demonstrating what motivations indicate positive and negative sexting expectancies. Individuals who sext for the purpose of engaging in sexual behaviors with the target of their sexting behavior have positive sexting expectancies. This both supports the assertions that sexting can be beneficial to an individual’s sex life [1,6,9] and useful for relationship therapists to consider as an intervention for individuals in relationships who are wanting to explore ways to increase sexual intimacy with their partner(s) [10,35,36].

However, the reverse appears to apply when an individual is motivated to sext for instrumental/aggressive reasons, thus predicting a negative sexting expectancy. These negative expectancies may be related to harming the relationship, damaging trust, or reducing the open communication that individuals experience regarding sexuality within their relationships. These are all important avenues for future researchers to consider exploring. Regardless of the application, finding that positive sext expectancies are related to those who sext for sexual motivations and negative sext expectancies are related to instrumental aggressive reasons furthers our understanding of why individuals engage in sexting. These findings help illuminate why some individuals may participate in unwanted consensual sexting [21], meaning an individual consents to sext with their partner even though they would prefer not to participate.

## Figures and Tables

**Table 1:** Bivariate Correlation Matrix.

	1.	2.	3.	4.	5.	6.	7.
1. SP	1						
2. IAR	0.09	1					
3. BIR	0.20 **	0.71 **	1				
4. PSES	0.56 **	0.04	0.12 *	1			
5. NSES	−0.02	0.66 **	0.51 **	−0.06	1		
6. PSER	0.40 **	0.13 **	0.20 **	0.70 **	0.10	1	
7. NSER	−0.09	0.65 **	0.51 **	−0.11 *	0.80 **	0.004	1

Note: * *p* < 0.05, ** *p* < 0.01; SP = Sexting Motivations—Sexual Purposes, IAR = Sexting Motivations—Instrumental/Aggravated Reasons, BIR = Sexting Motivations—Body Image Reinforcement, PSES = Positive Sexting Expectancies Sending, NSES = Negative Sexting Expectancies Sending, PSER = Positive Sexting Expectancies Receiving, NSER = Negative Sexting Expectancies Receiving.

## Data Availability

The data presented in this study are available on request from the corresponding author. The data is not publicly available as it is not a public data set.
